# Effects of COVID-19 Epidemic Lockdown on Postpartum Depressive Symptoms in a Sample of Italian Mothers

**DOI:** 10.3389/fpsyt.2020.589916

**Published:** 2020-11-17

**Authors:** Olivia Spinola, Marianna Liotti, Anna Maria Speranza, Renata Tambelli

**Affiliations:** Department of Dynamic and Clinical Psychology, Sapienza University of Rome, Rome, Italy

**Keywords:** COVID- 19, lockdown, postpartum depression (PPD), mental health, motherhood

## Abstract

The extraordinary health emergency of the COVID-19 pandemic represents a new challenge for mental health researchers and clinical practitioners. The related containment measures may be a risk factor for psychological distress and mood disorders, especially in at-risk populations. This study aims to explore the impact of COVID-19 on postpartum depressive symptoms in mothers with children below 1 year of age. An online questionnaire survey was therefore conducted in Italy between May and June 2020. The survey consisted of several self-administered questionnaires: besides some *ad-hoc* questionnaires, the Edinburgh Postnatal Depression Scale (EPDS), the Scale of Perceived Social Support (SPSS) and the Maternity Social Support Scale (MSSS) were used. Two hundred forty-three Italian women were included in the study. The prevalence of postpartum depression symptomatology among mothers was 44%, as measured through the EPDS (cut-off >12). Women who spent the isolation in northern Italy adopted maladaptive coping strategies significantly more than women living in areas at lower risk. The analysis highlighted a significant difference between the group that was not directly affected by the virus and women who have had a direct or indirect contact with it. Besides situational factors specific to the pandemic, the results show that there are some risk factors tied to the personal history of the mother (e.g., having had a previous abortion). These data should inform and enlighten future protocols of intervention.

## Introduction

Since its outbreak in the beginning of 2020, the COVID-19 epidemic has significantly impacted many countries, with Italy being one of the European nations most affected by it in terms of death-toll and diffusion. Italy has also undergone one of Europe's longest and most severe quarantines, which began at a National level on March the 9th and ended on May the 3rd. The restrictive measures set up to stop the virus have caused the shutdown of all non-essential businesses and services, including schools, daycares, universities, and almost any kind of workplace, forcing Italians to remain confined in their homes for 3 months. This has caused not only serious social isolation and the disruption of daily habits, but often a job loss or a reduction in financial income. These restrictive measures and their effects, on top of the loss of close ones and the indirect exposure to mass trauma through news and social media, have already been shown to have increased stress, fear, and anxiety among Italians, significantly affecting their psychological well-being, especially in the case of more vulnerable and/or at risks populations (1, 2).

On this subject, a recently published review by Brooks et al. (3) explored the psychological impact of quarantine. The authors identified some important stressors, which included longer quarantine duration, infection fears, frustration, boredom, inadequate supplies, inadequate information, financial loss, and stigma. As far as symptoms are concerned, the review indicates that studies that compared psychological outcomes for people quarantined with those not quarantined (4–6), found that the former were significantly more likely to report exhaustion, detachment from others, anxiety, irritability, insomnia, poor concentration and indecisiveness, deteriorating work performance, trauma-related mental health disorders and depressive symptoms. Likewise, quantitative studies that only surveyed those who had been quarantined (7–10) reported more psychological symptoms, such as emotional disturbance, depression, stress, irritability, insomnia, and post-traumatic stress symptoms. Overall, this data highlights the critic impact of quarantine and isolation on mental well-being. Many researches have pointed out an increase in depressive symptomatology during COVID-19 in the general population, and Davenport et al. (11) have specifically identified an increase in the likelihood of maternal depression, highlighting how mothers could be at a particularly high-risk of developing psychological distress.

Transition to maternity, and specifically gestation of the first child, has been identified as a crucial life event (12). The ease or difficulty in which the woman makes this transition significantly affects her marital relationship and her early interactions with the child (13). Most part of the research carried out on becoming a mother is based on the experience of having the first child. However, some studies (14, 15) pointed out that having additional children leads to similar periods of adjustment (13). Lack of experience and representations restructuring is not the only source of stress during this transition: it seems that the process of having a new member into a pre-existing system is onto itself a source of crisis requiring readjustment (12). Overall, the transition to parenthood encompasses psychological (16), neurobiological (17); and socio-relational adjustments (18, 19). In this context of redefinition, postnatal depression (PND) is a major parental mental health issue (20). Specific risk factors for the insurgence of PND have been identified. Antenatal depression has been found to be the strongest predictor of postpartum depression (21, 22). Likewise, lack of social support, poor marital relationships (21), stressful life events during pregnancy and after delivery (23), and prior negative pregnancy experiences, such as abortion or miscarriage, all have been found to strongly predict postpartum depression (18, 19).

The estimated prevalence of postpartum depression ranges from 6.5 to 12.9% or even higher in lower-income countries (24). Symptoms of postpartum depression often include sleep disturbance, anxiety, irritability, a feeling of being overwhelmed and an obsessional preoccupation with the baby's health and feeding. Suicidal ideation and worries about causing harm to the baby have also been reported (25). Because of assessment reliability issues, the best method for detecting postpartum depression remains controversial. The term “depression” has been widely used to refer not only to a continuum of depressive mood and psychological distress, but also for a definite diagnostic category (26). Some studies (27) highlighted a prevalence of the latter one of around 10%, while when self-report questionnaires are used the percentage of women with depressive symptomatology is much higher (between 20 and 30%). However, more recently Shorey et al. (28) conducted a review which didn't find a significant difference between prevalence of PPD as diagnosed through self-report questionnaires and clinical interviews. Therefore, screening for PPD through the Edinburgh Postnatal Depression Scale [EPDS; (29)], as recommended by the American Academy of Pediatrics, ultimately seems to be an accurate method of identifying postpartum depression.

Mothers suffering from postpartum depression have been shown to be less sensitive to their infants' needs (30). This has an impact on mother-infant bonding and may also reduce breastfeeding (31). Furthermore, early interaction interferences might impair the cognitive, behavioral, and social-emotional development and physical health of the child (32).

Some research specifically exploring the impact of COVID-19 on maternity has already been carried out. Recently, a study (33) showed an increase in the prevalence of postpartum depression in Chinese women who gave birth during the peak of the COVID-19 epidemic, as measured through the EPDS. Similar results were found in another Chinese research on pregnant women: they showed higher scores at EPDS, high levels of anxiety and a strong tendency to self-harm. These elements appeared to be mediated by the fear of COVID-19 infection (34). Another study carried out by Davenport et al. (11) on mothers who were pregnant or in the first year after delivery found that an EPDS score >13 was self-identified in 15% of respondents before the pandemic and in 40.7% during the pandemic. Also, moderate to high anxiety was identified in 29% of women before the pandemic and in 72% of women during the pandemic. Likewise, Vazquez-Vazquez et al. (35) conducted a study exploring breastfeeding practices during COVID-19 in women living in the UK aged ≥18 years with an infant ≤ 12 months of age. They found that lockdown has had an impact on maternal experiences, resulting in distress for many women, and that it affected breastfeeding practices.

So far, the vast majority of studies have been conducted abroad. Since Italy has been severely affected by the virus, there is need for further studies exploring the impact of it on the well-being of mothers. Identifying early risk factors of subsequent potentially dysfunctional interactions is crucial. This work aims to explore how a major critical event such as the one posed by the COVID-19 pandemic interacts with the delicate phase of transition to motherhood (be it for the first time or not).

More specifically, our hypotheses were the following: that during COVID-19 pandemic there would be a higher prevalence of post-partum depressive symptomatology than previously reported in literature; that the coping strategies adopted by each woman, as well as her perceived level of stress and her perceived social support would be correlated to the presence of a depressive symptomatology; and that among women who spent the isolation in the areas with higher rates of COVID-19 infection such symptomatology would be higher. We were also interested in exploring how some factors related both to the COVID-19 situation (such as an eventual loss of employment or fear of infection) and to the women's personal history could be correlated to the presence of depressive symptoms.

## Methods

### Design

This cross-sectional study was conducted online. A survey has been set up through Google Forms. The survey has been online from May 11 to June 6, 2020 and it took ~20–25 min to be completed. Participants could stop the survey at any time and withdraw from the study. Furthermore, participants could interact with the principal investigator of the study through email messages at any time during and after study participation.

The survey addressed women living in Italy at the moment of the lockdown, aged over 18 years, speaking Italian and having a child between 0 and 1 year through a multistep procedure: (1) email invitation to no-profit associations dealing with new mothers; (2) dissemination of the link through social media channels (Facebook, Twitter, Instagram) and the mailing lists of national post-partum depression associations; (3) official communication channels (e.g., University websites; websites of the associations directly involved in the management of postpartum period).

The invitation letter included information on study purposes and confidentiality. The provision of the informed consent was mandatory in order to start the survey.

### Measures

#### Content of the Survey

The survey was designed by the study team who has experience in the field of postpartum depression and mother-child interactions. Questions that could provoke or worsen psychological distress were avoided. The survey included an *ad hoc* schedule with the following sections:

1. socio-demographic characteristics;
2. COVID-19 related factors;
3. Personal history factors and information about pregnancy and childbirth;
4. Support after pregnancy.

Additionally, the survey included the following self-reported questionnaires: the Brief Coping Orientation to Problems Experiences [Brief-COPE; (36)], the Edinburgh Postnatal Depression Scale [EPDS; (29)], the Perceived Stress Scale [PSS; (37)], the Maternity Social Support Scale (38). Respondents' main socio-demographic characteristics, as well as data on their COVID-19 experience (loss of job, infection of themselves or of close others, fear of being infected, instructions about breastfeeding, etc.) was collected through an *ad hoc* schedule.

#### Demographic Characteristics of Mothers

Demographic characteristics of women who took part to the study, including age, educational level (secondary school, undergraduate degree, master's degree, Ph.D. or postgraduate title), profession (unemployed, student, housewife, freelancer, occasional job, employee, or other), weeks of the child, civil status and area of Italy of spent isolation were collected.

#### Personal History Factors

Personal history factors included having had previous abortions, having had childbirth complications, child's health at birth, having breastfed, having had other children and having had previous emotional troubles. Previous emotional troubles were assessed through the question: “Have you ever suffer from emotional troubles?,” to which a respondent could respond “yes” or “no”.

#### COVID-19 Related Factors

COVID-19 related factors included: loss of job; received support from family; infection of themselves; infection of close others; fear of being infected; fear of a close one being infected; fear of the child being infected; having received instructions about breastfeeding; believing that COVID-19 has affected breastfeeding.

#### Perceived Support After Pregnancy

Support after pregnancy was measured using the Maternity Social Support Scale [MSSS, (38)], a self-report questionnaire which consists of 6 items exploring relational factors commonly associated with postpartum depression (family support, support network, help from partner/spouse, conflict with partner/spouse, feelings of being controlled by partner/spouse, and feelings of being loved by partner/spouse), each rated on a 5-point Likert scale. Low scores at the MSSS are significantly related to poorer health conditions and to higher scores at instruments measuring post-partum depressive symptoms (38).

##### Measurement of Coping Strategies

Use of coping strategies was explored through the Brief COPE (36), a self-reported questionnaire which consists of 28 items involving questions about one's way of coping with stressful situations. Each item is answered on a four-point Likert scale. The questionnaire consists of 14 sub-scales: self-distraction; active coping; denial; substance use; use of emotional support; use of instrumental support; behavioral disengagement; venting; positive reframing; planning; humor; acceptance; religion; and self-blame. The scores obtained at each sub-scale are interpreted referring to two overarching coping styles: avoidant (denial, substance use, venting, behavioral disengagement, self-distraction, self-blame) and approach (active coping, positive reframing, planning, acceptance, seeking emotional support, seeking informational support). Although various categorization systems have been employed, the division in these two dimensions as different—and stable over time—strategies to cope with stress is one of the most utilized in mental health studies (39, 40). The humor and religion sub-scales aren't considered as part of neither an approach nor of an avoidance coping style. The relationships between coping strategies and maternal well-being has already been documented in literature: several studies have in fact found that the use of some particular coping strategies can differentiate between women with or without post-partum depressive symptoms; more specifically, the presence of avoidant coping strategies seem to be able to predict the development of depressive symptoms among mothers, both during pregnancy and after childbirth (41–45).

##### Measurement of Perceived Stress

The Perceived Stress Scale [PSS; (37)], was used to measure the perception of stress. This self-report instrument has a total of 10 items asking about one's feelings and thoughts in the past month. Each item is answered on a five-point Likert scale ranging from 0 (never) to 4 (very often). The overall score ranges from 0 to 40, with higher scores indicating higher levels of perceived stress. Scores between 0 and 13 are indicative of no stress, between 14 and 26 of stress, and between 27 and 40 of significant stress. The Cronbach's alpha coefficient for this scale in previous studies was 0.810 (46).

##### Measurement of Postnatal Depression

Depression symptoms were measured using the Edinburgh Postnatal Depression Scale [EPDS; (29)], a 10-item self-report scale designed to measure self-reported symptoms associated with depression experienced in the past week. Each item was scored using a four-points Likert scale (from 0 to 3). Scores were summed up, with 30 as the highest possible value. High values indicate strong symptoms. Mothers with scores of 13 or higher are regarded as likely to suffer from depression. This questionnaire is widely used to screen for depression, and has a Cronbach's alpha coefficient of 0.79; using a cut-off score of 12, it has good specificity (98.9%) and positive predictive value (90.9%) (47).

### Statistical Analysis

SPSS version 25 (IBM, Armonk, NY, United States) was used for analysis. Data are expressed as mean ± SD or number (percentage). All reported probability values for the *t*-test performed were 2-tailed, and the criterion for significance was set at *p* = 0.05. Descriptive statistics have been processed for the dependent and confounding variables.

Specifically, the analytic plan included: (1) data cleaning of the online dataset; (2) descriptive statistics of the general characteristics of the recruited sample, in terms of levels of depressive symptoms, coping strategies, perceived stress and social support, of closeness to infections and impact of COVID-19 on the socio-economic status, (3) sub-groups analyses based on the level of impact of the pandemic on postpartum depression symptoms. Namely *t*-tests were carried out to test for the effects of the following variables on global EPDS scores: Suspension from work (self or partner), Received economic support from family, Infected by the virus, Infection of close one, Contact with infected ones, Influence of COVID-19 on breastfeeding practices, Fear of being infected, fear of child being infected, Fear of close ones being infected, Previous Abortion, Birth Complications, Breastfeeding, Other children, Previous Emotional Troubles, Child's good health at birth. Two-way Analysis of Variance (2-way ANOVA) were ran in order to test for the interaction between fixed factors but no significant results were found therefore they were not included in the final version of the manuscript. Lastly, bivariate correlation between MSSS, PSS, EPDS were carried out, and criterion for significant correlation was set at *p* = 0.05.

## Results

### Characteristics of the Sample

The sociodemographic characteristics and clinical features of respondents are shown in Table 1. Two invalid records were removed. Two hundred forty-three women were included in the study (Age M = 34 years old, range= 21–47; sd = 4.27). Inclusion criteria were: having a child between 0 and 52 weeks of age, being in Italy during the lockdown. Regarding the area in which subjects have been during the lockdown, 53.9% of the sample has been isolated in northern Italy, and 44.90% in central or southern Italy. On average, at the moment of the research the child age was 14.79 weeks old (range = 0–48; sd = 9.12), and 93.4% of women was married or co-living.

**Table 1:** Sociodemographic characteristics of the sample.

**Characteristics**	**Subgroup**	**Value**	***N***
**Mean age, y**	–	34.01	243
**Mean child age, wk**	–	15.11	235
**Area of Italy (spent isolation, %)**	North	53.90	131
	Center and South	44.90	109
**Level of education (%)**	Secondary school	18.85	46
	Undergraduate degree	19.80	48
	Master's degree	34.84	85
	Phd/Postgraduate title	26.30	64
**Civil status (%)**	Single	5.33	13
	Married/Cohabitating	93.44	227
	Divorced/Separated	0.80	2
	Widowed	0.41	1
**Profession (%)**	Unemployed	10.30	25
	Student	2.50	6
	Housewife	3.70	9
	Occasional job	2.90	7
	Freelancer	19.8	48
	Employee	55.6	136
	Other	5.30	13

As far as personal history data is concerned, results showed that 65% of the sample was having the first child, and 32.1% had had previous abortion. Furthermore, 12.3% of women suffered from previous chronic diseases, and 28.4% declared to have suffered from previous emotional problems.

Regarding situational factors (inherent to the COVID-19 pandemic), 21.0% of the sample had one or more close person infected from the virus, and 3.7% of the women who took part to the survey had been infected. Approximately 62.6% of the sample was afraid of being infected, 83.1% feared that a closed one such as their partner could be infected, and 84% of women was afraid that their children could be infected. More than 72% of the sample has had a suspension of the work (own's or partner's job), and 21.0% received economical support from the family.

### Standardized Questionnaires Global Scores

The most striking result concerns the EPDS total scores (see Table 2 for detailed scores and frequencies). More than 44% of the sample has a score above the cut-off (≥12) for postpartum depression symptomatology.

**Table 2:** Self-report scales cut-off for clinical significance and % of the sample above cut-off.

**Self-report scale**	***N***	**Cut-off**	**% over cut-off**	**Min-Max**	**Mean score**	**Standard deviation**
Edinburgh postnatal depression scale (EPDS)	243	>12	44.40	0–26	10.86	6.37
Perceived stress scale (PSS)	243	>27	51.90	14–39	26.65	5.80
Maternity social support scale (MSSS)	243	<18	87.20	6–29	11.51	4.75

Moreover, 51.90% of the sample had a score above the cut-off for significant stress perceived (>27), and 87.20% of the sample had a perceived Maternal Social Support of <18, indicative of very low support perceived.

### Impact of COVID-19 Related Variables on Depressive Symptomatology

Several *t*-tests were ran so to explore the effects of COVID-19 related variables on the prevalence of depressive symptomatology (means, *p*-values, *t* scores and standard deviations are shown in Table 3). Results pointed out a statistically significant effect of having had a suspension from work (own's or other's) and of having received economic support from the family. Also, having been in touch with the COVID-19 had a significant impact on the EPDS scores. More specifically, having been infected by the virus, having had a close one infected or having been in contact with infected ones all had a significant effect on the total score of the EPDS. Likewise, fear for the child being infected also had a significant effect on the postpartum depressive symptomatology as measured through the EPDS. Surprisingly, women who reported not to be afraid of close ones being infected exhibited higher scores at EPDS scores compared to women who stated to have such fears.

**Table 3:** Effects of COVID-19 related variables on Edinburgh Postnatal Depression Scale (EPDS) total score.

**Characteristics**		***N***	**Mean**	**Standard deviation**	***T***	***p***
Suspension from work (self or partner)	Yes	175	11.50	6.31	2.55	**0.01**
	No	68	9.21	6.27		
Received economic support from family	Yes	192	12.61	6.25	2.22	**0.05**
	No	51	10.43	6.34		
Infected by the virus	Yes	9	18.00	7.05	3.5	**0.001**
	No	234	10.59	6.20		
Infection of close one	Yes	51	13.69	6.80	3.65	**0.000**
	No	192	10.11	6.05		
Contact with infected ones	Yes	220	16.00	6.45	4.16	**0.000**
	No	23	15.96	6.59		
Influence of COVID-19 on breastfeeding	Yes	96	14.55	5.97	8.25	**0.000**
	No	147	8.45	5.41		
Fear of being infected	Yes	152	11.09	5.90	0.733	n.s.
	No	91	10.47	7.11		
Fear of child being infected	Yes	204	11.59	6.29	4.24	**0.000**
	No	39	7.03	5.41		
Fear of close ones being infected	Yes	202	10.37	5.97	−2.71	**0.01**
	No	41	13.29	7.72		

*Bold values indicates the significant values*.

### Effects of Personal Variables on Depressive Symptomatology

As far as effects of personal characteristics on the self-report scales are concerned, the *t*-test analysis highlighted that the presence of one or more abortions in women's previous history has a significant effect on depressive symptomatology, with women having had a previous abortion showing higher scores on the EPDS compared to that presented by women without previous abortions.

Likewise, having had previous emotional problems also had a significant effect on the EPDS scores. Having other children, birth complications, breastfeeding practices and child's health at birth didn't have a significant effect on the scores at the different scales. (Tables 4, 5).

**Table 4:** Effects of women's personal characteristics on Edinburgh Postnatal Depression Scale (EPDS) total score.

**Characteristic**		***N***	**Mean**	**Standard deviation**	**t**	***P***
Previous abortion	Yes	78	13.64	6.14	4.84	**0.001**
	No	165	9.59	6.07		
Birth complications	Yes	63	11.95	6.44	1.58	n.s.
	No	180	10.48	6.32		
Breastfeeding	Yes	197	10.73	6.16	−0,65	n.s.
	No	46	11.41	7.25		
Other children	Yes	85	10.53	6.14	−0.64	n.s.
	No	158	11.04	6.50		
Previous emotional troubles	Yes	69	12.74	6.20	2.94	**0.001**
	No	174	10.11	6.30		
Child's good health at birth	Yes	219	10.84	6.32	−0.126	n.s.
	No	24	11.04	6.94		

*Bold values indicates the significant values*.

**Table 5:** Areas of Italy and BRIEF-COPE avoidant strategies.

**Sub-scale**	**Area**	***N***	**Mean**	**Standard deviation**	***t***	***p***
Substance abuse	Center and South	109	4.78	1.49	−2.50	**0.01**
	North	131	5.18	1.45		
Self distraction	Center and South	109	4.78	1.49	−2.21	**0.04**
	North	131	5.17	1.45		
Denial	Center and South	109	3.12	1.43	−0.65	n.s.
	North	131	3.23	1.33		
Venting	Center and South	109	5.08	1.76	−0.48	n.s.
	North	131	5.18	1.44		
Behavioral disengagement	Center and South	109	2.97	1.27	−0.32	n.s.
	North	131	3.02	1.17		
Self blame	Center and South	109	5.08	1.45	−0.72	n.s.
	North	131	5.23	1.35		
Total avoidant style	Center and South	109	23.17	4.46	−1.83	n.s.
	North	131	24.17	4.01		

*Bold values indicates the significant values*.

### Correlation Between Self-Report Scales and EPDS Scores

Correlational analysis were ran for continuous variables (see Table 6 for Pearson correlation values and significance). Results showed a statistically significant positive correlation between the scores at the EPDS and SPS scores, avoidant strategies as measured through the BRIEF-COPE scales and MSSS scores. Statistically significant negative correlations were found between EPDS scores and approaching strategies (measured through the BRIEF-COPE).

**Table 6:** Correlation of edinburgh postnatal depression scale (EPDS) score with perceived stress scale (PSS), maternal social support scale (MSSS) and brief coping orientation to problems experiences (BRIEF-COPE) scores.

**Self-report scale**	***r***	***p***
Perceived stress scale (PSS)	0.774	**0.001**
Maternity social support scale (MSSS)	0.410	**0.001**
BRIEF-COPE (Avoidant Strategies)	0.356	**0.001**
BRIEF-COPE (Approaching Strategies)	−0.135	0.037

*Bold values indicates the significant values*.

### Effect of Area of Spent Isolation

In order to further explore the specific impact of the area of isolation (north vs. center and south) the sample was divided into two groups depending on the area where they spent the isolation (North: Piemonte, Liguria, Veneto, Friuli Venezia Giulia, Trentino Alto Adige, Emilia Romagna e Lombardia) Center and South (Lazio, Toscana, Puglia, Abruzzo, Basilicata, Calabria, Campania, Sicilia, e Marche). A *t*-test was then carried out to see whether there was a difference in terms of EPDS scores between northern areas and central and southern ones. We decided to divide the sample in these two groups, considering central and southern areas as a whole, because in those areas the risk of contagion and the diffusion of the virus was similar, and significantly lower than in the north (48).

Results pointed out a significant effect of the area of isolation on total EPDS scores, with northern areas having higher means (see Table 7 for means and Standard Deviations). A similar result was found for the scores at the Perceived Stress Scale, with northern areas exhibiting significantly higher stress scores.

**Table 7:** Areas of spent isolation and Edinburgh Postnatal Depression Scale (EPDS) scores.

**Area**	***N***	**Mean**	**Standard**	***t***	***p***
			**deviation**		
North	131	11.85	6.56	−2.50	0.01
Center and South	109	9.82	5.94		

Lastly, we wanted to understand whether there was a difference in terms of coping strategies between northern areas of Italy and central and southern. We therefore carried out further *t*-tests which highlighted a significant difference between these areas only for the scales measuring Substance Abuse and Self Distraction, both being part of dysfunctional coping strategies (see Table 8 for means and Standard Deviations).

**Table 8:** Areas of spent isolation (South, Center and North of Italy) and PSS scores.

**Area**	***N***	**Mean**	**Standard**	***t***	***p***
			**deviation**		
North	132	27.77	5.62	−3.01	0.003
Center and South	109	25.58	5.62		

## Discussion

This study investigated the impact of COVID-19 pandemic on the psychological well-being of mothers of children aged from 0 to 1 year old. An EPDS score of >12 (indicative of postpartum depression symptomatology) was self-reported in 44% of the sample. Likewise, a perceived stress of >27 (indicative of a substantial stress perceived) was self-reported in 43.4% of the sample. These findings illustrate a significant increase in depressive symptomatology and stress in mothers during the pandemic compared to the self-reported rates of depressive symptomatology in the general population.

Several COVID-19 related variables showed to have a significant effect on the EPDS scores. Specifically, a significant effect of fear of infection (for others and child) on EPDS scores was found. Women who reported to be scared of having their own child infected, reported higher levels of depressive symptomatology compared to women who didn't state to have such fears. This is coherent with the results of a recent study published by Dsouza et al. (49) about the causative factors of COVID-19 related suicide-incidence. The authors found fear of COVID-19 infection to be the prominent causative factor of COVID-19 related suicides, and they hypothesize a relationship with the lack of literacy and the presence of a stigma around mental health in rural areas in India, where the study was carried out. This is an important result because it suggests that a lack of appropriate information about the COVID-19 could play an important role in increasing levels of fear, anxiety, depression and other symptoms. However, it is important to notice that women who reported not to be afraid of close ones being infected exhibited higher scores at EPDS compared to women who didn't report such fears. It could be speculated that use of strategies such as denial of anxious internal states may increase the depressive symptomatology, but the sole use of a self-report scale to measure depressive symptomatology calls for further exploration of the afore-mentioned effect.

Relevantly, in our survey some women reported to have received indication of breastfeeding their child with the mask during the COVID-19 pandemic by their physicians. This kind of information might had contributed to fear of infection and of infecting the child. In fact, a striking result is that women did not report fear of infection for themselves, but only for their children or significant others around them. Coherently with this hypothesis, another factor that had a significantly influence on the EPDS scores in our study was indeed the belief that COVID-19 has affected breastfeeding practices, reported by around 61% of women in our sample.

Besides, having had a close one infected by the virus also significantly increased the EPDS scores. This influence appears rather intuitive, since having close ones infected is likely to increase fear of loss and psychological burden of the pandemic. Also having been in contact with someone who had been infected increased the self-reported post-depression symptomatology. Having been in touch with a COVID-19 positive person might have increased the fear of infecting the child and of being harmful to the child, thus lessening the self-efficacy and sense of adequacy as a new mother.

Regarding the socio-economic impact of COVID-19 on the mother, a decrease in income of the mother or of the father of the child was significantly associated with higher scores at the EPDS. This result is in concordance with an earlier review conducted which found decreased socioeconomic status to significantly increase the risk for postpartum depression (50).

Interestingly, receiving economic support from the family significantly increased the EPDS scores. We can only speculate about what causes women receiving economical support to experience higher symptoms of postpartum depression. One possible explanation could be that receiving support affects the perception of one's self as competent and self-efficient, and therefore reduces the self-esteem of the new mother.

Not surprisingly, women who spent the isolation in northern areas of Italy—which have been most severely affected by the virus in terms of death toll and contagions—reported higher levels of postpartum depression symptomatology and of perceived stress than women who spent the isolation in central or southern areas of the country.

Beyond COVID-19 related factors, some specific characteristics belonging to the personal history of the mother also had an effect on the depressive symptomatology. Namely, having had a previous abortion significantly increased the likelihood of developing postpartum depression. The role of previous abortion as a potential risk factor for the onset of postpartum depression, despite being quite intuitive, has not been extensively documented in the literature. A review from Hamama (51) found a rate of PTSD during the subsequent pregnancy of women who had an abortion of 12.6%, and the rate of depression was 16.8%, as assessed through phone clinical interviews. However, the detailed analysis of the association of past abortions with subsequent pregnancy mental health status indicated that it was not the experience of abortion itself that increased the risk of PTSD or depression, but the appraisal of the abortion as having been a hard time (i.e., potentially traumatic) that predicted subsequent morbidity. Despite there being existing evidence showing the association between abortion and increase in risk of mental disorders (52), further research is needed to elucidate the specific mechanisms underlying the effects of previous abortions on subsequent pregnancies. This is a fundamental aspect to control for since it represents a potential risk factor for increased vulnerability of the mother.

Having had previous psychiatric history also had a significant effect on the EPDS total scores, with women having a history of emotional troubles exhibiting higher scores. This is a risk factor which has already been documented in the literature (53), hence highlighting the importance of a correct screening procedure including questions relating to previous psychiatric history. Another interesting finding was observed regarding coping strategies. Significant differences between northern and central/southern areas were found for the scales of Substance abuse and Self-distraction, both part of the avoidant strategies, higher in the north. Previous studies have already found that an avoidant coping strategy is related to more symptoms of depression (40). More specifically, women with passive coping strategies (i.e., with higher scores on the denial, behavioral disengagement, self-blame and substance abuse scales) have been found to be more at risk of developing symptoms of antenatal and/or postnatal depression (41–45). Exploring the link between coping styles, post-traumatic stress and depressive symptoms in a sample of new mothers, another study found that self-distraction strategies were positively correlated with a depressive symptomatology (54). Targeting coping strategies in the intervention could be therefore particularly useful to reduce the depressive symptomatology in at-risk mothers.

Overall, the results of this study have a clinical relevance. Even though clinical diagnosis through psychiatric interviews remain elective, this study remarks a significant postpartum depressive symptomatology increase in women with children below 1 year. Throughout periods of confinement and of restrictions, follow-up should therefore be as close as possible, and the administration of a questionnaire specifically exploring the presence of personal and situational risk factors could be extremely useful. If postpartum depression is a multifaceted disorder which results from a dynamic interaction between biological, psychological, and social risk factors, the current COVID-19 pandemic likely has amplified them all (55). The severe consequences of postpartum depressive symptomatology on the mother-infant bonding and global development of the child are now widely documented in the literature (56) and calls for a thorough and specific attention. Having identified some of the potential risk factors associated with the onset of a pandemic is of crucial importance so to inform clinical practices and develop prevention programs aiming to support this at-risk population.

## Limitations and Future Directions

### Limitations

There are several caveats to consider when interpreting the results of this study. First of all, women having pre-existing interest in mental health or experiencing distress might have been more likely to respond to take part to this survey, thus potentially rendering the sample not fully representative of the general population. Furthermore, past studies highlighted that the quality of response in online surveys tends to be reduced (57). This is even more problematic considering the small size of the sample of this survey, which might contribute to an underpower of the statistically significant results of this study. Another limitation resides in the use of a self-report instrument to evaluate the presence of a post-partum depressive symptomatology among women, such as the EPDS. Although the questionnaire has proven to be effective to identify the presence of PPD (28), an actual diagnosis could only be obtained through a clinical interview performed by a licensed health-care professional. Lastly, the correlational nature of the study doesn't allow to establish a causal relationship between the variables but only permits to infer correlations between them.

### Future Directions

The long-term significance of these findings for mother-infant interactions and subsequent outcomes still has to be explored. Since a large part of the sample gave its consent to be contacted for a second part of the study, future studies could investigate the impact of COVID-19 on mother-child interactions and on child's global development in a longitudinal perspective. A further question to be explored is whether these COVID-19-related depressive symptoms are somehow comparable to depressive states of other nature.

## Data Availability Statement

The raw data supporting the conclusions of this article will be made available by the authors, without undue reservation.

## Ethics Statement

The studies involving human participants were reviewed and approved by Ethics Committee of Sapienza Università di Roma. The patients/participants provided their written informed consent to participate in this study.

## Author Contributions

The data were collected by OS, and ML, while data analysis was conducted by AS, OS and ML. All authors study was designed contributed, writing of the manuscript, read and approved the final manuscript.

## Conflict of Interest

The authors declare that the research was conducted in the absence of any commercial or financial relationships that could be construed as a potential conflict of interest. The reviewer, DA, declared a past co-authorship with one of the authors, RT, to the handling Editor.
